# Oncostatin M induces heat hypersensitivity by gp130-dependent sensitization of TRPV1 in sensory neurons

**DOI:** 10.1186/1744-8069-7-102

**Published:** 2011-12-23

**Authors:** Michiel Langeslag, Cristina E Constantin, Manfred Andratsch, Serena Quarta, Norbert Mair, Michaela Kress

**Affiliations:** 1 Division of Physiology, Department of Physiology and Medical Physics, Medical University Innsbruck, Innsbruck, Austria; 2 Institute of Physiology, University of Freiburg, Freiburg, Germany

**Keywords:** inflammation, inflammatory pain, proinflammatory cytokines, heat hypersensitivity, transsignaling

## Abstract

Oncostatin M (OSM) is a member of the interleukin-6 cytokine family and regulates eg. gene activation, cell survival, proliferation and differentiation. OSM binds to a receptor complex consisting of the ubiquitously expressed signal transducer gp130 and the ligand binding OSM receptor subunit, which is expressed on a specific subset of primary afferent neurons. In the present study, the effect of OSM on heat nociception was investigated in nociceptor-specific gp130 knock-out (*SNS-gp130^-/-^*) and gp130 floxed (*gp130^fl/fl^*) mice.

Subcutaneous injection of pathophysiologically relevant concentrations of OSM into the hind-paw of *C57BL6J wild type *mice significantly reduced paw withdrawal latencies to heat stimulation. In contrast to *gp130^fl/fl ^*mice, OSM did not induce heat hypersensitivity *in vivo *in *SNS-gp130^-/- ^*mice. OSM applied at the receptive fields of sensory neurons in *in vitro *skin-nerve preparations showed that OSM significantly increased the discharge rate during a standard ramp-shaped heat stimulus. The capsaicin- and heat-sensitive ion channel TRPV1, expressed on a subpopulation of nociceptive neurons, has been shown to play an important role in inflammation-induced heat hypersensitivity. Stimulation of cultured dorsal root ganglion neurons with OSM resulted in potentiation of capsaicin induced ionic currents. In line with these recordings, mice with a null mutation of the TRPV1 gene did not show any signs of OSM-induced heat hypersensitivity *in vivo*.

The present data suggest that OSM induces thermal hypersensitivity by directly sensitizing nociceptors via OSMR-gp130 receptor mediated potentiation of TRPV1.

## Background

Oncostatin M (OSM) is a monomeric glycoprotein that belongs to the interleukin-6 (IL-6) family of proinflammatory cytokines. Besides OSM, this family consists of IL-6, interleukin-11 (IL-11), leukemia inhibitory factor (LIF), ciliary neurotrophic factor (CNTF) and cardiotrophin-1 [1]. OSM is produced by several cell types of the immune system, including activated monocytes and T-cells [2], macrophages [3], eosinophils and polymorphonuclear neutrophils [4,5]. The physiological functions of OSM are divers, ranging from cell type specific proliferative or anti-proliferative effects and maturation of fetal hepatocytes to regulation of the inflammatory response.

In general, IL-6 related cytokines bind to their specific receptor and requires dimerization with the common signal transducer gp130 to form a functional receptor complex [6]. Whereas IL-6 binds to its receptor IL-6R and dimerizes with two molecules of gp130, OSM receptor (OSMR) signaling requires just a single gp130 molecule to exert its effects. Human OSM is capable of binding not only to the OSMR but also to the LIF receptor, in contrast to murine OSM that only binds to the OSMR [7]. A subset of murine afferent sensory nerve fibers expresses OSMR. These OSMR-positive neurons also express the heat receptor TRPV1 and the purinergic receptors that are well recognized mediators in pain perception [8]. Studies in *OSM *knock-out mice showed that OSM has a profound role in the development of a particular subtype of nociceptors. The absence of OSM leads to a substantial decrease in the number of OSMR-positive neurons [9]. Moreover, OSM-deficient mice displayed reduced nociceptive behavior to acute noxious mechanical, thermal and chemical stimuli.

During inflammation, release of inflammatory mediators and pro-inflammatory cytokines occurs and in elevated levels of OSM are found in patients with rheumatoid arthritis up to 1 ng/ml in synovial fluid [10,11]. In addition, acute inflammation of the skin by complete Freund's adjuvant results in elevated levels of OSM, but expression of OSMR in dorsal root ganglia remains unaltered [12]. Inflammatory mediators cause hypersensitivity to noxious heat and mechanical stimuli of the inflamed tissue. Members of the IL-6 cytokine family are involved in chronic pain during chronic inflammation like rheumatoid arthritis and this is at least partially due to the sensitization of primary nociceptive afferents innervating the inflamed tissue. IL-6/gp130 receptor-mediated signaling in Nav1.8 expressing nociceptive afferents induces increased heat sensitivity both *in vitro *and *in vivo *through direct regulation of TRPV1. *In vitro*, gp130-deficient nociceptors are unresponsive to IL-6 stimulation with regard to sensitization of TRPV1. Furthermore, mice lacking gp130 in nociceptors display reduced levels of pain perception induced by inflammation and tumor growth [13].

Since ligand binding induces OSMR heteromerization with gp130 and OSMR-positive neurons coexpress TRPV1 in mice, we set out to determine the effect of OSM on heat nociception in nociceptor-specific gp130 knock-out (*SNS-gp130^-/-^*) and gp130 floxed (*gp130^fl/fl^*) mouse strains. Our results demonstrate that OSM induces heat hypersensitivity by directly sensitizing heat nociceptive neurons via receptor-mediated potentiation of ionic currents that are induced by the TRPV1 stimulant capsaicin. Furthermore, we find that the subset of OSM-sensitive nociceptors seems to contribute significantly to heat hypersensitivity.

## Results

To investigate whether OSM can induce heat hypersensitivity dependent on OSMR-gp130 receptor activation, in this study we used sensory neuron specific (SNS) knock out of the signal transducer gp130. Small and medium sized nociceptive sensory neurons exclusively express the sodium channel Nav1.8 and its promotor has been used to specifically express the Cre-recombinase in sensory neurons (*SNS-Cre*). Mice homozygous for the floxed allele of the mouse gp130 gene (*gp130^fl/fl^*) were crossed with SNS-Cre mice to create *SNS-gp130^-/- ^*[13].

### Oncostatin M Receptor β and signal transducer gp130 are co-expressed in DRG neurons and potentiate capsaicin-activated currents

**Figure 1:**
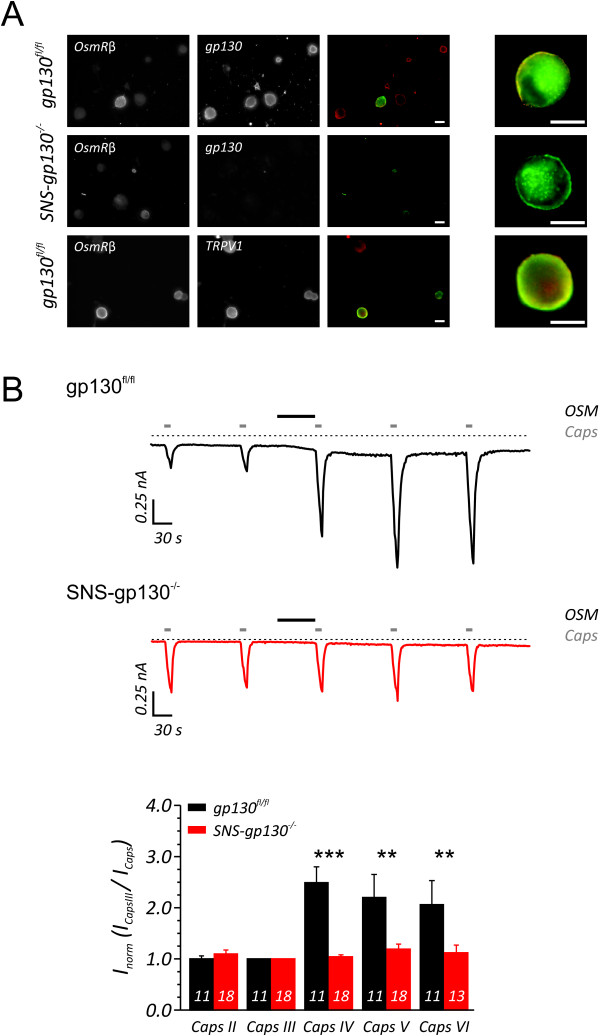
**OSM requires the expression of gp130 in nociceptive neurons to potentiate capsaicin-evoked currents**. (A) Expression of OSMR, gp130 and TRPV1 in *gp130^fl/fl ^*and *SNS-gp130^-/- ^*cultured DRG neurons. OSMR and gp130 immunoreactivity was observed in *gp130^fl/fl ^*DRG neurons (upper panel). In addition, the OSMR-positive neurons of *gp130^fl/fl ^*mice also displayed TRPV1 immunoreactivity (middle panel). The *SNS-gp130^-/- ^*DRG neurons were devoid of gp130 (lower panel) while maintaining OSMR expression. Scale bar 10 μM. (B) In electrophysiological recordings of *gp130^fl/fl ^*neurons OSM (5 ng/ml) induces a transient potentiation of capsaicin-responsive currents (upper trace), whereas in DRG neurons lacking the gp130 expression OSM did not increase the capsaicin-induced currents (lower trace). Quantification of normalized ionic currents (I_caps_/I_capsIII_) reveals that OSM increases capsaicin currents approximately 2.5 fold within 2 minutes in OSMR-positive gp130 nociceptors compared to the normalized currents recorded in OSMR-positive/gp130-negative *SNS-gp130^-/- ^*nociceptive neurons. ** p < 0.01, *** p < 0.001, Mann-Whitney U-test, white numbers inside the individual bars indicate the number of recorded Icaps.

For OSM to exert its' action on DRG neurons, both the OSMR and gp130 should be expressed in the same neuron. Live-cell labeling of *gp130^fl/fl ^*neurons against the OSMR and gp130 revealed that all OSMR-positive cells (n = 27) express gp130 (Figure 1A, upper panel). To elucidate whether OSM directly affects heat sensitivity of sensory neurons, mice with a conditional deletion of gp130 (*SNS-gp130^-/-^*) and control *gp130^fl/fl ^*[13] were used. In neurons isolated from *SNS-gp130^-/- ^*mice, OSMR-positive neurons lacked detectable levels of full length gp130 at the plasmamembrane as observed by live-cell labeling (n = 41, Figure 1A, middle panel). Furthermore, we found that all OSMR-positive in both *gp130^fl/fl ^*(Figure 1A, lower panel) and *SNS-gp130^-/- ^*(data not shown) neurons also express TRPV1 and is in accordance to previous findings [8]

The nociceptor-specific heat- and capsaicin-sensitive ion channel TRPV1 is essential for the induction of inflammatory pain and hyperalgesia [14,15]. There is an expanding amount of evidence that inflammatory mediators are capable of regulating TRPV1 activity and this process is essential in the generation of heat hypersensitivity. In a cellular model of nociception, modulation of TRPV1 can be addressed by recording capsaicin-induced currents (I_caps_) in small diameter neurons. To examine whether OSM causes acute and direct modulation of heat-sensitive neurons, we challenged the neurons with 5 ng/ml OSM (similar to previous studies [16-18] and comparable to OSM levels found in chronic RA patients [11]). Patch-clamp experiments were carried out on isolated DRG neurons of adult *gp130^fl/fl ^*and *SNS-gp130^-/- ^*mice and were immuno-labeled against both OSMR and gp130. In all OSMR-positive cells that were recorded, repetitive application of capsaicin evoked reproducible inward currents of relatively constant amplitude. Challenging the *gp130^fl/fl ^*DRG neurons with OSM resulted in a marked potentiation of subsequent I_caps _stimulations in all neurons investigated (Figure 1B, top trace); maximum amplitudes of I_caps _increased by a factor of 2.49 ± 0.31 (Figure 1C, black bars). When OSMR-negative/gp130-positive neurons were recorded and stimulated with OSM, no potentiation of I_caps _was observed (n = 3, data not shown). In OSMR-positive/gp130-negative neurons obtained from *SNS-gp130^-/-^*, OSM induced augmentation of I_caps _was absent (Figure 1B, lower trace, Figure 1C, red bars).

### The signal transducer gp130 mediates Oncostatin M-induced nociceptor sensitization *in vitro*

**Figure 2:**
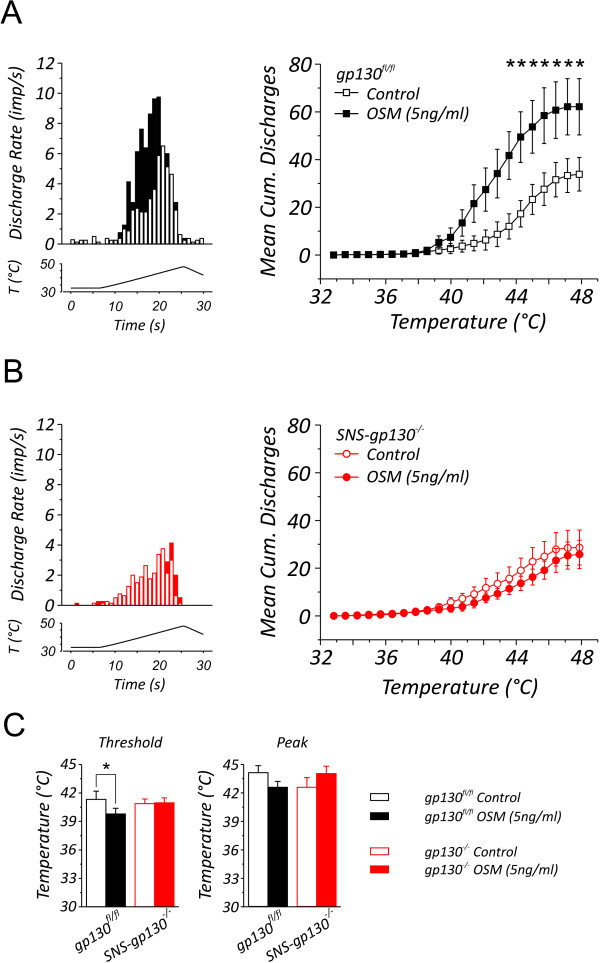
**Discharge characteristics of heat-sensitive C-fibers innervating the dorsal site of the hindpaw recorded in an *in vitro* skin-nerve preparation from *gp130^fl/fl ^*and *SNS-gp130^-/- ^*mice upon a heat-ramp stimulus**. (A) *Left panel*; the discharge pattern of *gp130^fl/fl ^*C-fibers is changed upon stimulation with 5 ng/ml OSM, the amount of discharges recorded is increased and has shifted to lower temperatures. The mean cumulative discharge rates in heat-sensitive C-fibers from *gp130^fl/fl ^*were significantly higher after perfusion with 5 ng/ml OSM (filled squares, n = 8) compared with rate of discharge before OSM application (open squares, n = 8). (B) However, in *SNS-gp130^-/- ^*C-fibers, the discharge pattern evoked by a heat ramp stimulus is unaltered after stimulation with OSM (*left panel*). Furthermore, OSM perfusion failed to increase the mean cumulative discharge rate of heat-sensitive fibers in *SNS-gp130^-/- ^*skin-nerve preparations (control; open circles, +OSM; filled circles, n = 8). (C) The discharge threshold temperature of *gp130^fl/fl ^*(n = 8,) is significantly decreased after stimulation with OSM (5 ng/ml), whereas in *SNS-gp130^-/- ^*the threshold temperature remained unchanged (left bar graph, n = 8). The temperature at which the maximal discharge rate was achieved was unaltered in both *gp130^fl/fl ^*and *SNS-gp130^-/- ^*(right bar graph, n = 8). * p < 0.05, Wilcoxon signed rank test.

In order to address the question whether OSM can also sensitize nociceptive primary afferents within innervated tissue, we performed single fiber recordings from unmyelinated, heat-sensitive primary afferents in a skin nerve *in vitro *preparation [19,20]. Polymodal nociceptors from *gp130^fl/fl ^*responded to a standard ramp-shaped heat stimulus with an average discharge rate of 2.19 ± 0.44 imp/s (n = 8) (Figure 2A, left panel, open bars). At the end of the heat stimulus, the heat-sensitive fibers from *gp130^fl/fl ^*responded with a total of 33.9 ± 7.0 action potentials (Figure 2A, right panel, open squares). The nociceptive fibers started to respond at 41.3 ± 0.9°C (Figure 2C, left graph, black open bar) and the maximal amount of action potentials generated by the *gp130^fl/fl ^*fibers was recorded at an average temperature of 44.1 ± 0.74°C (Figure 2C, right graph, black open bar). Exposing the receptive fields from *gp130^fl/fl ^*preparations to a conditioning OSM (5 ng/ml) stimulus, the average discharge rate increased to 3.86 ± 0.76 imp/s during the consecutive heat stimulus (Figure 2B, left panel, black bars) and the total number of discharges is significantly increased to 62.1 ± 11.8 (Figure 2A, right panel, closed squares). In addition, the threshold temperature to which fibers started generating action potentials is significantly decreased (Figure 2C, left graph, 39.79 ± 0.6°C, p < 0.05). Besides, the average temperature at which the highest discharge rate was recorded shifted non-significantly to slightly lower temperatures (42.6 ± 0.6°C, Figure 2C, right graph).

In preparations from *SNS-gp130^-/- ^*mice, the heat-sensitive fibers reacted with the highest discharge frequency at an average temperature of 42.6 ± 1.0°C (Figure 2C, right graph) and during the heat stimulus a total of 28.6 ± 7.3 action potentials were recorded (Figure 2B, right graph, open circles) with an average discharge rate of 1.43 ± 0.25 imp/s (Figure 2B, left graph, open bars). In contrast to the *gp130^fl/fl ^*preparations, OSM did not enhance heat responses in *SNS-gp130^-/- ^*preparations (Figure 2B, right graph, closed circles; total discharges per fiber: 25.9 ± 6.0, Figure 2B, left graph, red bars; average discharge rate: 1.29 ± 0.25 imp/s). Neither was the threshold temperature altered by OSM (Figure 2C, left graph before; 40.9 ± 0.5°C, after; 41.0 ± 0.5°C) nor was the average temperature at which the highest discharge rates were recorded (44.0 ± 0.78 imp/s, Figure 2C, right graph).

### Oncostatin M induces hypersensitivity to heat stimuli *in vivo*

**Figure 3:**
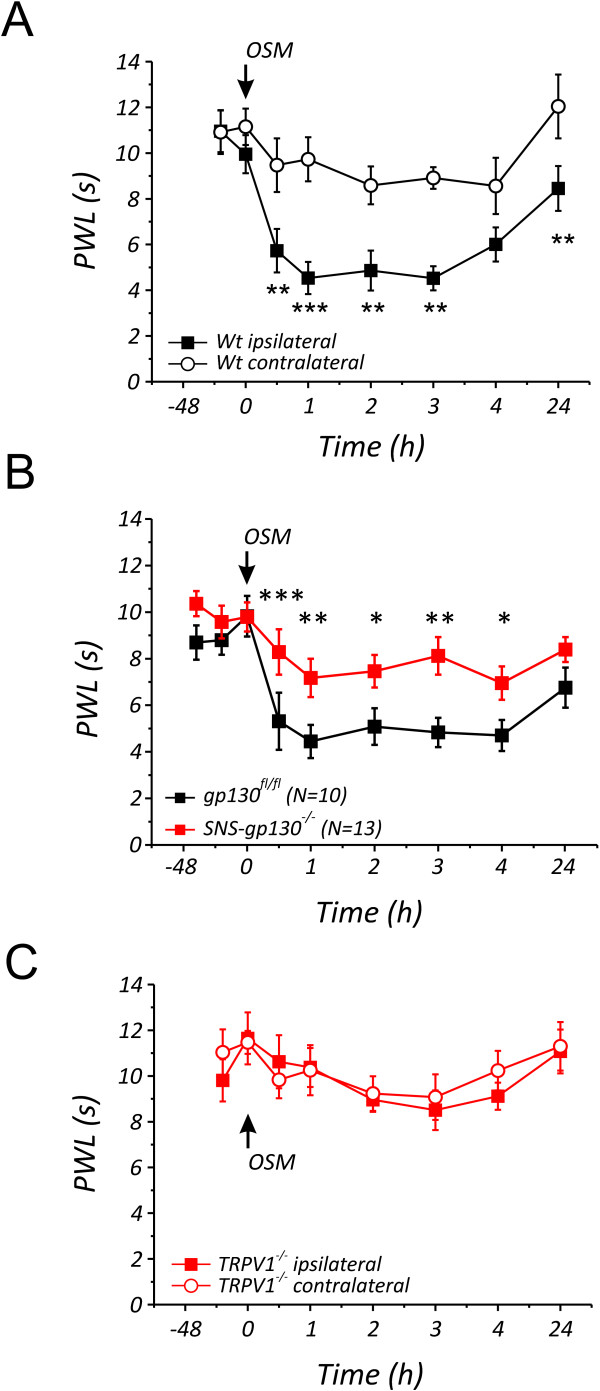
**OSM induces thermal hypersensitivity depending on gp130 and TRPV1**. (A) Changes in heat sensitivity following hindpaw injection of OSM in *C57BL6J wildtype *mice. Paw withdrawal latencies were determined at the injected (black squares) and the uninjected hindpaw (black open circles) in response to ramp-shaped heat stimuli and were significantly attenuated at all time points at the ipsilateral site compared to contralateral site (n = 10). (B) Comparison of the PWL between both *gp130^fl/fl ^*and *SNS-gp130^-/- ^*mice at the OSM injected hindpaw shows a significant difference; the PWL of *gp130^fl/fl ^*(black squares, n = 10) are significantly shorter than those of *SNS-gp130^-/- ^*(red squares, n = 13) starting after 30 minutes until 4 hours after OSM injection. (C) In contrast, the PWL in response to ramp-shaped heat stimuli applied to the plantar side of the hindpaw ipsilateral (red open circle) and contralateral (red squares) of *TRPV1^-/- ^*mice (n = 11) did not significantly change after OSM injection. * p < 0.05, ** p < 0.01, *** p < 0.001, 2-way repeated ANOVA.

To investigate the relevance of the present findings *in vivo*, heat sensitivity was assessed in mice using the Hargreaves test before and after subcutaneous injection of OSM. Injection of 10 ng/10 μl OSM reduced paw withdrawal latencies (PWL) in *C57BL6J wild type *(*wt*) mice at the ipsilateral side from 9.95 ± 0.79s to 5.73 ± 0.90s at the first measurement 30 minutes after injection compared to the contralateral side. This hypersensitivity to heat remained significant for 24 hours after injection (Figure 3A). To address the importance of gp130 for OSM-induced heat hypersensitivity *in vivo*, *SNS-gp130^-/- ^*and *gp130^fl/fl ^*were used in behavioral tests. OSM-induced heat hypersensitivity was observed after 30 min. in *gp130^fl/fl ^*(Figure 3B, PWL 9.93 ± 0.87s before vs. 4.44 ± 0.72s after OSM) and remained substantially different for 4 hours compared to the contralateral side (data not shown). Similar to *wt *mice, the PWL of *gp130^fl/fl ^*mice remained significantly different up to 24 hours after injection of OSM in the hindpaw. Injection of the vehicle alone in the hindpaw did not cause any changes in heat sensation [21]. In *SNS-gp130^-/- ^*mice, there was no significant decrease of the paw withdrawal latency (Figure 3B, from 9.80 ± 0.63s to 7.17 ± 0.82s) observed after subcutaneous injection of OSM (10 ng/10 μl) compared to the uninjected contralateral site (9.66 ± 0.78s to 7.41 ± 0.76s, data not shown). The OSM-induced decrease of PWL in *gp130^fl/fl ^*was already significantly reduced 30 minutes after injection compared to the PWL of *SNS-gp130^-/- ^*mice (Figure 3B). Behavioral studies performed on motor pathways in both *gp130^fl/fl ^*(n = 8) and *SNS-gp130^-/- ^*(n = 8) mice using the rotarod test did not show any differences between the 2 mouse strains (data not shown). These results strongly suggest that the signal transducer gp130 in nociceptors is essential in mediating OSM induced heat hypersensitivity in mice.

During inflammatory conditions, the increased heat hypersensitivity is dependent on TRPV1, and TRPV1-deficient mice do not develop heat hypersensitivity [22]. Since OSM can be secreted during inflammation by T lymphocytes [2] and neutrophils [4], we examined whether OSM would induce heat hypersensitivity in mice with a global deletion of TRPV1 (*TRPV1^-/-^*). Intracutaneous injection of OSM into the plantar hindpaw of *TRPV1^-/- ^*mice did not result in a notable change in heat sensitivity compared to baseline values or to the contralateral side (Figure 3C). Furthermore, there was no significant difference observed between the PWLs of *wt *and *TRPV1^-/- ^*mice at the contralateral side (data not shown).

## Discussion

Our data show OSM induced thermal hypersensitivity via direct regulation of nociceptor sensitivity to heat stimuli. Furthermore, the gp130 signal tranducer, which heteromerizes with the OSMR to form a functional receptor complex, is of critical importance in mediating the OSM-induced heat hypersensitivity in mice. This is justified by the observation that nociceptors from *SNS-gp130^-/- ^*mice are largely protected from OSM-induced heat sensitization and by the absence of OSM induced potentiation of I_caps _in gp130-deficient sensory neurons. Furthermore, we show that the TRPV1 ion channel is the convergence point for thermal hypersensitivity mediated by Oncostatin M.

Proinflammatory cytokines including OSM that depend on gp130 for signal transduction require the presence of specific ligand binding, soluble or membrane bound, receptor subunits as well as the presence of gp130 signal transducer to transduce the signal into the cytosol [23,24]. The majority of nociceptive neurons in mice express gp130 and can be activated by classical transsignaling routes via soluble receptors, e g. for IL-6 [25]. Therefore, activation of specific DRG neuron subpopulations can only be discriminated by different expression patterns for specific membrane bound receptors. Approximately 13% of the DRG neurons of *C57BL6 wild type *mice express the OSMR and it is most prevalent in small-sized neurons [26]. Furthermore, all OSMR-positive neurons express the signal transducer gp130 and classical nociceptive markers TRPV1 and P2X3 receptors. Typically, the OSMR expressing neurons are non-peptidergic, but are rather characterized by expression of TrkA (63%), Ret (28%) and/or IB4 (58%). Our observation that all OSMR positive cells derived from *C57BL6J *or *gp130^fl/fl ^*DRG neurons are co-expressing gp130 is in agreement with these previous findings [26]. As expected, all OSMR-positive neurons were responsive to capsaicin in our electrophysiogical recordings and responded to OSM with a potentiation of I_caps_.

Oncostatin M has an important role in the development of nociceptive neurons. The OSMR expression is induced after p0 in the DRGs and reaches maximal expression after p14. The kinetics of OSMR expression is not altered in *OSM^-/- ^*mice, however the total number of OSMR-positive cells is dramatically reduced. Moreover, the number of TRPV1 and/or P2X3 positive neurons is even further decreased, resulting in decreased nociceptive behavior upon various nociceptive models [9]. Our data obtained from *SNS-gp130^-/- ^*mice in behavioral studies and skin-nerve preparations similarly show decreased OSM-induced nociceptive behavior to heat. The PWL in Hargreaves tests are not reduced after OSM injection neither is the discharge rate in skin-nerve preparations after OSM-perfusion. Undoubtedly, according to our results, OSM and the OSM receptor are important mediators of thermal hypersensitivity of preexisting polymodal nociceptors in adult mice.

Furthermore, our behavioral studies show that both gp130 and TRPV1 are required for developing OSM-induced heat hypersensitivity. Previously, our lab presented data that IL-6 increases heat sensitivity of nociceptors through modulation of TRPV1 and is completely dependent on gp130. Both OSM and IL-6 require gp130 for signal transduction, OSMR dimerizes with a single gp130 molecule whereas the IL-6R associates with two gp130 molecules [27,28]. Although, the intracellular signal transduction pathways are quite similar, both will activate Shp2-Gab1 and the JAK/STAT signaling pathway, with the only difference that OSMR can activate STAT1, -3, and -5, whereas IL-6 only activates STAT1 and -3 [29,30]. However, the heat hypersensitivity induced by IL-6 relies on TRPV1 modulation via Gab1, PI3K and PKCδ [13]. Since, the time course of OSM-induced potentiation of capsaicin currents is very similar to the IL-6 mediated potentiation of TRPV1 currents in *gp130^fl/fl ^*and *wildtype *DRGs we therefore assume that OSMR/gp130 uses the same pathway for heat sensitization as we have previously published for IL-6/gp130.

OSM and the OSMR are well-known players during various inflammatory conditions. For example, OSM is a potent cytokine that induces cutaneous inflammation after intradermal injection in mice, and results in neutrophil infiltration [31]. Besides, skin and breast tissue inflammation is associated with psoriasin (S100A7) expression, which is potently increased by OSM in human breast cancer cells *in vitro *[32]. Moreover, many (auto-) inflammatory diseases are related to OSM, like rheumatoid arthritis [10], atherosclerosis [33], psoriasis and atopic dermatitis [2]. Oncostatin M has a pivotal role in rheumatoid arthritis (RA), where increased levels of OSM are found in the synovial fluid of RA patients. Synovial fibroblasts are stimulated to proliferate and to produce IL-6 by OSM [34]. These findings combined with the fact that RA coincides with increased mechanical and thermal sensitivity [35,36], we assume that inhibiting OSM signaling by a novel strategy with soluble gp130 [6,37] or by a soluble OSMR-gp130 fusion protein [38] would effectively reduce OSM-induced hypersensitivity in RA.

Although expression of OSM and OSMR has been reported previously and a role for OSM has been shown for nociceptor development, our data for the first time demonstrate the function of OSM in inducing thermal hypersensitivity in mice. Our data show that OSM potentiates I_caps _and sensitizes nociceptors via regulation of TRPV1. Although we cannot rule out the OSM mediated indirect influence of immune cells, like neutrophils and macrophages, or keratinocytes in *in vivo *experiments, we have provided genetic evidence that OSM acts directly on capsaicin-responsive nociceptive neurons via the common gp130 signal transducer expressed in sensory neurons.

## Materials and methods

### Genetically modified mice

*SNS-gp130^-/- ^*and *gp130^fl/fl ^*mice were generated, bred and genotyped as described previously [13]. All mice were maintained under SPF (specific pathogen free) conditions. Littermates were used in all experiments to control for background effects and all animal use procedures were in accordance with ethical guidelines and animal welfare standards according to Austrian law. All behavioral measurements were done in awake, unrestrained, age-matched male mice that were > 8 weeks old by individuals who were blinded to the genotype of the mice being analyzed.

### Primary sensory neuron culture

All lumbar (L1-L6) dorsal root ganglia (DRG) containing the cell bodies of primary afferents that project into the hindpaw were harvested from adult mice as previously published [13,39]. After removal of the connective tissue, ganglia were incubated in Liberase Blendzyme 1 (9 mg/100 ml DMEM, Roche) for 2 times 30 min. After washing with PBS (PAA), 1x Trypsin-EDTA (Invitrogen) was added for 15 min. and DRG were washed with TNB™ medium (Biochrom) supplemented with L-glutamin (Invitrogen), penicillin G sodium, streptomycin sulfate (Invitrogen), and Protein-Lipid-Komplex™ (Biochrom). The DRG were dissociated with a fire-polished Pasteur pipette and centrifuged through a 3.5% BSA gradient (Sigma) to eliminate non-neuronal cells. The sensory neurons were resuspended, plated on coverslips coated with poly-L-Lysine/laminin-1 (Sigma), and cultivated in supplemented TNB™ containing mNGF 2.5S (Alomone Labs, 10 μg/100 ml TNB-medium) at 37°C in 5% CO_2 _for 24-36 h.

### Immunocytochemistry and live-cell labeling

DRG neurons were dissociated according to the protocol and plated on coverslips. After 24 h in culture, neurons were fixed with 4% PFA for 20 min, permeabilized with 0.01% TX-100 (Sigma) for 2 min and blocked with blocking buffer (BB, 10% goat serum in PBS) for 30 min. Cells were incubated with primary antibodies against OSMR (MLB) and TRPV1 (Millipore) in BB (1:50 and 1:1000 respectively) for 1 h at room temperature (RT), washed with PBS, and incubated with secondary antibodies (1:1000) for 30 min at RT.

Overnight DRG neuron cultures were live cell labeled for gp130 and/or OSMR expression shortly before recording. Cultures were incubated on RT with primary antibodies OSMR and gp130 (Neuromics) diluted in TNB™ medium (1:25 and 1:50 respectively) for 45 min, washed with TNB™ medium for 10 min, and incubated with appropriate secondary antibodies (AlexaFluor-488 and -594 conjugated, Invitrogen) diluted in TNB™ medium (1:500) for 30 min. Subsequently cells were washed twice with TNB™ medium.

The TRPV1 antibody used does not stain murine *TRPV1^-/- ^*DRG neurons as shown previously [13]. Furthermore, *SNS-gp130^-/- ^*DRG neurons do not display gp130 staining at the plasmamembrane when live labeled, while *gp130^fl/fl ^*neurons do [13]. The OSM antibody used only stained approximately 13% of non-permeabilized cultured neurons with a clear plasmamembrane staining whereas the remainder of the neurons did not. Besides, when OSM-negative/gp130-positive neurons were recorded and stimulated with OSM, no potentiation of I_caps _was observed (n = 3), providing specificity of the OSM antibody.

### Patch-clamp recordings

DRG neurons in culture were used for electrophysiology 18 to 28 h after plating. Ionic currents were recorded from isolated neurons in the whole-cell voltage-clamp configuration of the patch-clamp technique as previously published [13,21,39]. ECS contained (in mM): 150 NaCl, 5 KCl, 0.1 CaCl_2_, 1 MgCl_2 _(all Sigma), 10 glucose, and 10 HEPES (Merck), at pH 7.3 adjusted with NaOH (Merck). Calcium was reduced to 0.1 mM to avoid desensitization of capsaicin-induced currents. Borosilicate glass pipettes (Science Products) pulled with a horizontal puller (Sutter Instruments Company) were filled with internal solution (ICS) (in mM): 148 KCl, 2 MgCl_2_, 2 Na-ATP, 0.1 CaCl_2_, 1 EGTA (all Sigma), and 10 HEPES (Merck), at pH 7.3 adjusted with KOH (Merck). After filling, electrode resistance was 4-5 MΩ. Neurons were clamped at -80 mV holding potential. Currents were sampled at 3 kHz and filtered at 2.9 kHz, and recorded with an EPC 9 (HEKA) and the Pulse v8.74 software (HEKA). A seven barrel system with common outlet was used for fast drug administration (WAS 02, Dittel, Prague). Capsaicin-activated inward currents (I_caps_) were elicited by applying 0.5 μM capsaicin (Sigma) for 10 s followed by a 2 min washout with control solution. Capsaicin stimulation was repeated 3 times before OSM (5 ng/ml in ECS, R&D systems) was applied for 1 min immediately before capsaicin for conditioning stimulation. Experiments were performed at room temperature and only one neuron was tested per Petri dish.

### Skin-nerve preparation and single fiber recordings

An *in vitro *skin-nerve preparation was used to investigate the properties of the afferent nerve fibers innervating the skin of the mouse dorsal hindpaw as previously published [19,20]. Briefly, the preparation was superfused (15 ml/min) with an oxygen-saturated modified synthetic interstitial fluid solution containing (in mM) 108 NaCl, 3.48 KCl, 3.5 MgSO_4_, 26 NaHCO3, 1.7 NaH_2_PO_4_, 2.0 CaCl_2_, 9.6 sodium gluconate, 5.5 glucose, 7.6 sucrose at temperature of 31 ± 1°C, and pH7.4 ± 0.05. Action potentials of single sensory neurons were recorded extracellularly from fine filaments dissected from the saphenous nerve, amplified (5000-fold), filtered (low pass 1 KHz, high pass 100 Hz), visualized on oscilloscope, and stored on a PC-type computer with Spike/Spidi software package [40]. The fibers were characterized as unmyelinated (C) according to their conduction velocity (c.v.< 1.4 m/s) as calculated from the latency of the action potential electrically evoked at the receptive field and the distance between receptive field and recording electrode. The receptive field was identified by mechanical probing of the skin with a glass rod; standard heat stimuli linearly rising the intracutaneous temperature from 31 ± 1°C to 47°C were applied. A fiber was considered heat sensitive if 3 or more action potentials were evoked during the stimulus. Afterwards the C-fibers were challenged with OSM (5 ng/ml in synthetic interstitial fluid solution) for 5 minutes and subsequently recorded during a second heat stimulus. The heat threshold was defined as the temperature that elicited the third spike of the response.

### Behavioral testing

Standard testing procedures were used to quantify signs of nociceptive behavior. The area tested was the plantar side of the hindpaw. Heat sensitivity was assessed using the Hargreaves test [41]. Paw withdrawal latency in response to an increasing radiant heat stimulus was measured automatically (Ugo Basile) before and after OSM (10 ng/ml, PBS) injection.

### Statistical analysis

For detailed statistical analysis the Origin 7.0 (Originlab) and Sigmastat 3.0 (Aspire Software International) were used. Data are presented as mean ± SEM and were analyzed using the Wilcoxon signed rank test, Mann Whitney-U test or two-way repeated-measures ANOVA for comparison between groups and test days. Differences were considered statistically significant at *p *< 0.05.

## List of abbreviations

OSM: Oncostatin M; OSMR: Oncostatin M receptor; LIF: leukemia inhibitory factor; IL-6: Interleukin-6; gp130: glycoprotein 130; TRPV1: transient receptor potential vanilloid 1; SNS: sensory neuron specific; DRG: dorsal root ganglia; I_caps_: capsaicin-activated inward current; PWL: paw withdrawal latency; RT: room temperature; PBS: phosphate buffered saline; DMEM: Dulbecco's modified Eagle medium; BSA: bovine serum albumin; PFA: paraformaldehyde

## Competing interests

The authors declare that they have no competing interests.

## Authors' contributions

CEC and NM performed patch-clamp electrophysiology. CEC performed behavioral studies and single fiber recording. MA was responsible for generating the *SNS-gp130^-/- ^*mice, breeding and genotyping of the mice strains. SQ performed immunohistological stainings. ML and MK designed and finished the final draft of the manuscript. All authors read and approved the final manuscript.
